# Diagnostic Performance of Carotid Contrast-Enhanced Ultrasound for Identifying Functionally Significant Coronary Artery Stenosis Assessed by Quantitative Flow Ratio: A Preliminary Prospective Study

**DOI:** 10.3390/jcdd13050202

**Published:** 2026-05-09

**Authors:** Yuehao Song, Jili Long, Hao Wang

**Affiliations:** 1 Department of Echocardiography, State Key Laboratory of Cardiovascular Disease, Fuwai Hospital, National Center for Cardiovascular Diseases, Chinese Academy of Medical Sciences and Peking Union Medical College, Beijing 100037, China; b2025002067@student.pumc.edu.cn; 2 Department of Ultrasound, The Second Affiliated Hospital of Chongqing Medical University, Chongqing 400010, China; longjili2020@163.com

**Keywords:** carotid artery, contrast-enhanced ultrasound, quantitative flow ratio, coronary artery stenosis, plaque vulnerability

## Abstract

**Background:** Carotid contrast-enhanced ultrasound (CEUS) provides a noninvasive means of assessing plaque vulnerability and may reflect the systemic burden of atherosclerosis. This study aimed to evaluate the diagnostic performance of carotid CEUS characteristics for identifying functionally significant coronary artery stenosis (CAS) defined by quantitative flow ratio (QFR). **Methods:** In this preliminary prospective study, 46 patients with suspected stable coronary artery disease who underwent carotid CEUS and coronary angiography with QFR assessment between September 2022 and November 2023 were enrolled. Patients were categorized into a QFR ≥ 0.80 group (n = 18) and a QFR < 0.80 group (n = 28). Carotid plaque burden, morphology, and CEUS-derived quantitative parameters were compared between groups. Univariate and multivariable logistic regression analyses were performed to identify independent factors associated with QFR < 0.80, and receiver operating characteristic (ROC) analysis was used to assess discriminatory performance. **Results:** Compared with patients with QFR ≥ 0.80, those with QFR < 0.80 had significantly higher mean intima-media thickness (IMT), larger plaque area, higher plaque-to-lumen enhancement ratios (Pmax/Cmax and Pmean/Cmean), and more vulnerable plaque features, including irregular margins and thin fibrous caps. In multivariable analysis, Pmax/Cmax (adjusted OR: 14.394, 95% CI: 2.718–76.220; *p* = 0.002) and mean IMT (adjusted OR: 7.740, 95% CI: 2.040–29.363; *p* = 0.003) remained independently associated with QFR < 0.80. ROC analysis showed that the combined model incorporating Pmax/Cmax and mean IMT achieved the best discrimination for QFR < 0.80 (AUC: 0.931, 95% CI: 0.845–0.989), with 78.6% sensitivity and 94.4% specificity. **Conclusions:** Carotid CEUS-derived plaque enhancement characteristics, particularly Pmax/Cmax, together with mean IMT, were independently associated with functionally significant CAS. These findings suggest that carotid CEUS may provide complementary, noninvasive information for vascular risk stratification, but it should not be considered a substitute for coronary angiography-based physiological assessment.

## 1. Introduction

Atherosclerotic cardiovascular disease remains the leading cause of cardiovascular mortality worldwide [1]. Atherosclerosis is a systemic disorder that affects multiple vascular parts, including the coronary arteries [2]. Coronary artery stenosis caused by lipid deposition and plaque formation impairs myocardial perfusion and may lead to ischemia and infarction, representing the central pathological feature of coronary artery disease (CAD) [3,4]. For patients with CAD, accurate and early identification of functionally significant coronary artery stenosis (CAS) is crucial for appropriate treatment selection and improved prognosis [5]. Carotid contrast-enhanced ultrasound (CEUS), as an important tool for cardiovascular risk stratification, may serve as a noninvasive window reflecting systemic atherosclerotic burden and plaque vulnerability.

Fractional flow reserve (FFR) has long been regarded as the gold standard for evaluating the hemodynamic significance of coronary stenosis [6]. However, its invasive nature, the need for pressure wire instrumentation and hyperemic agents, longer procedural time, and higher cost limit its widespread clinical use. Quantitative flow ratio (QFR), an angiography-based functional assessment technique, enables computation of coronary flow physiology without a pressure wire or pharmacologic hyperemia [7]. Existing evidence has shown that QFR has high diagnostic accuracy for identifying functionally significant coronary stenosis and demonstrates good agreement with FFR (typically using QFR ≤ 0.80 as the reference threshold) [8,9].

Previous studies have suggested that carotid CEUS may predict functionally significant coronary stenosis and overall CAD risk, primarily by assessing intraplaque neovascularization as a surrogate of systemic plaque vulnerability [10]. The degree of CEUS plaque enhancement has been associated with severe coronary lesions and adverse cardiovascular outcomes and may offer better risk stratification than common carotid intima-media thickness in some settings while adding prognostic value beyond traditional risk factors [11,12]. Although the association between carotid CEUS findings and coronary events has been reported, more comprehensive studies are still needed to clarify the diagnostic value of carotid CEUS parameters for identifying functionally significant coronary stenosis.

Therefore, this preliminary prospective study aimed to evaluate the diagnostic performance of carotid CEUS characteristics for identifying functionally significant CAS, defined as QFR <= 0.80. By investigating the relationship between carotid CEUS parameters and coronary functional significance, we sought to explore the potential role of carotid CEUS as a noninvasive imaging window for cardiovascular risk assessment.

## 2. Methods

### 2.1. Study Population

This preliminary prospective study enrolled patients with suspected CAD based on clinical symptoms, cardiovascular risk factors, and abnormal noninvasive test findings. The indication for coronary angiography was determined by treating cardiologists based on the overall clinical assessment. Noninvasive testing was performed when clinically indicated but was not mandatory for all patients. Between September 2022 and November 2023, a total of 46 patients with stable CAD were included. Exclusion criteria were: age <18 years, baseline regional wall motion abnormalities, prior myocardial infarction, prior coronary artery bypass grafting, unstable angina, second- or third-degree atrioventricular block, chronic obstructive pulmonary disease, severe valvular heart disease, severe ventricular arrhythmia, severe hepatic or renal dysfunction, history of allergy to ultrasound contrast agents, prior intracardiac shunt surgery, pregnancy, and lactation.

All patients underwent carotid CEUS during the same period before coronary angiography and QFR assessment. According to QFR values, patients were categorized into a QFR >= 0.80 group and a QFR < 0.80 group (functionally significant CAS group). Baseline characteristics included hypertension, diabetes mellitus, smoking history, and drinking history. Resting systolic blood pressure and heart rate were measured, and carotid ultrasound examinations were performed in all participants.

All participants or their legal representatives provided written informed consent. The study complied with the Declaration of Helsinki (2013 revision) and was approved by the Institutional Review Board of the Ethics Committee of Fuwai Hospital (protocol code: No. 2021-1429 and date of approval: 26 January 2021).

### 2.2. Carotid Contrast-Enhanced Ultrasound (CEUS)

Carotid CEUS was performed using a Philips EPIQ 7C ultrasound system equipped with an L9-3 linear transducer (4–9 MHz). SonoVue was used as the contrast agent, prepared with 5 mL saline. Patients were examined in the supine position. Conventional carotid ultrasound was first performed to assess the common carotid artery, carotid bifurcation, and internal carotid artery bilaterally, including the proximal and distal segments as much as possible.

Maximum plaque height was defined as the maximum distance from the intima–lumen interface to the media–adventitia interface after comparing both carotid artery walls. For each patient, the plaque with the greatest maximum plaque height was selected for analysis. After target plaque selection, CEUS mode was activated. A bolus of 2.4 mL contrast agent was injected via the cubital vein, followed immediately by 5 mL saline flush, and dynamic images were continuously recorded for 5 min.

A region of interest (ROI) was delineated within the plaque, and a reference ROI was placed in the adjacent arterial lumen. Time–intensity curve (TIC) analysis was performed to derive quantitative parameters, including AUC, wash-in slope, time to peak, peak intensity, time from peak to half, and rise time. Enhancement ratios were calculated using plaque ROI and lumen ROI measurements.

Carotid plaque grading was performed using an ultrasound/contrast-enhanced ultrasound-adapted Plaque-RADS approach, with reference to the Carotid Plaque-RADS framework proposed by Saba et al. [13]. CEUS was used to semiquantitatively assess intraplaque neovascularization (IPN) in carotid plaques. According to the Chinese expert consensus and previous CEUS studies, a 3-grade system was first applied as follows: grade 0, no visible enhancement within the plaque; grade 1, limited enhancement/microbubbles confined to the plaque shoulder and/or adventitial side; and grade 2, extensive intraplaque enhancement with microbubbles extending into the plaque core. In addition, a 4-grade enhancement-distribution system was used: grade 1, no enhancement within the plaque; grade 2, enhancement at the plaque base; grade 3, enhancement at the base and shoulder; and grade 4, enhancement involving the base, shoulder, and internal/core region of the plaque.

The “fibrous cap” was also evaluated. In this study, the term “fibrous cap” was used as an ultrasound-based operational descriptor rather than a direct histopathological measurement. On conventional carotid ultrasound, the fibrous cap is defined as the echogenic layer covering the luminal surface of the plaque. A visually thin or attenuated fibrous cap was recorded when this luminal-side echogenic layer appeared thin, indistinct, or discontinuous. Because ultrasound cannot resolve microscopic fibrous cap thickness, this parameter should be interpreted as an imaging surrogate of plaque surface morphology.

All CEUS images were independently reviewed by two experienced vascular sonographers who were blinded to the clinical data, and disagreements were resolved by consensus. All images were analyzed in a blinded manner by two experienced sonographers.

### 2.3. Coronary Angiography and Online QFR Assessment

Coronary angiographic images were visually interpreted by an experienced cardiologist blinded to ultrasound data. Two angiographic projections separated by at least 25 degrees were transmitted through the local network to the QFR analysis system [14] (AngioPlus, Shanghai Pulse Medical Technology Co., Ltd., Shanghai, China), which computes QFR using a previously described algorithm. After 3-dimensional reconstruction of the coronary arteries, QFR values were obtained for the left anterior descending artery (LAD), left circumflex artery (LCX), and right coronary artery (RCA). The lowest QFR value among LAD, LCX, and RCA was used as the final patient-level QFR value.

**Figure 1:**
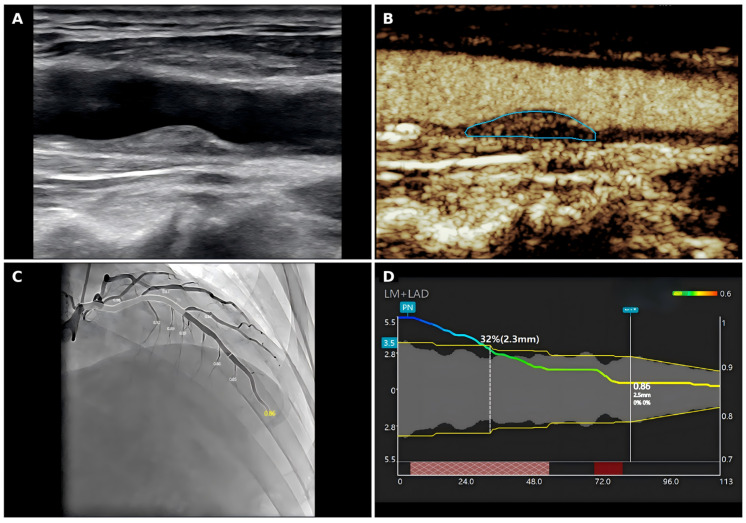
Representative carotid ultrasound, carotid CEUS, and QFR images in a patient with functionally significant coronary artery stenosis. (**A**) Conventional carotid ultrasound showing carotid plaque morphology. (**B**) Carotid contrast-enhanced ultrasound showing plaque enhancement and the selected plaque region. (**C**) Coronary angiography image of the LAD. (**D**) QFR analysis curve showing the corresponding functional assessment. CEUS, contrast-enhanced ultrasound; LAD, left anterior descending artery; QFR, quantitative flow ratio.

Two trained medical technicians, blinded to clinical and CEUS findings, independently performed QFR measurements of clinically relevant lesions, and the average of their measurements was used for analysis. Functionally significant CAS was defined as QFR <= 0.80.Representative images of conventional carotid ultrasound, carotid CEUS, coronary angiography, and QFR analysis in a patient with functionally significant CAS are shown in Figure 1.

### 2.4. Statistical Analysis

Continuous variables were expressed as mean +/− standard deviation (SD) or median with interquartile ranges. Categorical variables were presented as counts and percentages. Normality was assessed using the Shapiro–Wilk test. Between-group comparisons were performed using the Student’s *t*-test or Mann–Whitney U test for continuous variables, and categorical data were analyzed using the chi-squared test or Fisher’s exact test.

To identify factors associated with functionally significant CAS (QFR < 0.80), variables with statistically significant between-group differences (*p* < 0.05) were included in univariate screening. Continuous variables were reported as odds ratios (ORs) per 1-SD increase. For categorical variables with sparse cells, overall association *p* values from contingency table analyses (chi-square/Fisher’s exact test) were used for screening. After excluding collinear variables based on correlation analysis and model parsimony considerations, representative variables were entered into the multivariable logistic regression model.

Receiver operating characteristic (ROC) curve analysis was performed using QFR as the reference standard to evaluate the discriminative performance of selected CEUS-related parameters and the multivariable model-derived probability. A two-sided *p* value < 0.05 was considered statistically significant. Statistical analyses were conducted with SPSS version 25.0 (IBM, Armonk, NY, USA), MedCalc, version 22.0 (MedCalc Software Ltd., Ostend, Belgium).

As this was a preliminary exploratory prospective study, no formal a priori sample-size calculation was performed. The final sample size was determined by the number of consecutive eligible patients who underwent both carotid CEUS and coronary angiography with QFR assessment during the study period.

## 3. Results

### 3.1. Patient Characteristics

A total of 46 participants were included in the final analysis, all of whom underwent both carotid CEUS and QFR assessment. According to QFR, 18 patients were assigned to the QFR >= 0.80 group and 28 patients to the QFR < 0.80 group. Baseline clinical characteristics are summarized in Table 1. QFR < 0.80 showed a significantly higher proportion of male patients than QFR ≥ 0.80 (92.9% vs. 66.7%, *p* = 0.042).

### 3.2. Carotid CEUS and Plaque-Related Characteristics

Compared with the QFR ≥ 0.80 group, patients with QFR < 0.80 had significantly higher average IMT (1.04 ± 0.12 vs. 0.92 ± 0.15, *p* = 0.011), larger plaque area (21.41 [16.31, 29.05] vs. 13.68 [9.64, 18.24] mm^2^, *p* = 0.003), higher Pmax/Cmax (0.75 [0.64, 0.84] vs. 0.44 [0.21, 0.67], *p* < 0.001), and higher Pmean/Cmean (0.61 ± 0.22 vs. 0.37 ± 0.26, *p* = 0.003).

In addition, significant between-group differences were observed in several plaque morphology-related variables, including maximum plaque echogenicity pattern (*p* = 0.033), margin characteristics (*p* = 0.004), fibrous cap (FC) category (*p* = 0.006), and Plaque-RADS classification (*p* = 0.036), and the QFR < 0.80 group showed a higher proportion of irregular margins and thin fibrous caps. Detailed comparisons of quantitative plaque parameters, luminal CEUS parameters, plaque morphology, and CEUS grading variables between the two QFR groups are presented in Table 2.

### 3.3. Univariate and Multivariable Logistic Regression Analysis

Using QFR < 0.80 as the dependent variable, univariate analysis demonstrated that the following continuous variables were significantly associated with functionally significant CAS (OR per 1-SD increase): Pmax/Cmax (OR: 4.143, 95% CI: 1.743–9.849, *p* = 0.001), Pmean/Cmean (OR: 2.887, 95% CI: 1.395–5.977, *p* = 0.004), average IMT (OR: 2.519, 95% CI: 1.215–5.221, *p* = 0.013), and plaque area (OR: 2.950, 95% CI: 1.050–8.290, *p* = 0.040). The results of univariate screening for factors associated with QFR < 0.80 are shown in Table 3.

For multivariable logistic regression, representative continuous variables were selected based on statistical significance and collinearity considerations. The final model included Pmax/Cmax and average IMT. Both remained independently associated with QFR < 0.80: Pmax/Cmax (adjusted OR: 14.394, 95% CI: 2.718–76.220, *p* = 0.002) and average IMT (adjusted OR: 7.740, 95% CI: 2.040–29.363, *p* = 0.003).The multivariable logistic regression results are presented in Table 4.

### 3.4. ROC Analysis for Identifying QFR < 0.80

ROC analyses were performed for the continuous variables retained after between-group screening. Pmax/Cmax, Pmean/Cmean, average IMT, and plaque area (Area) all demonstrated discriminative ability for identifying QFR < 0.80. The AUCs were 0.813 (95% CI: 0.673–0.928) for Pmax/Cmax, 0.756 (95% CI: 0.592–0.899) for Pmean/Cmean, 0.735 (95% CI: 0.579–0.888) for average IMT, and 0.766 (95% CI: 0.617–0.896) for plaque area.

**Figure 2:**
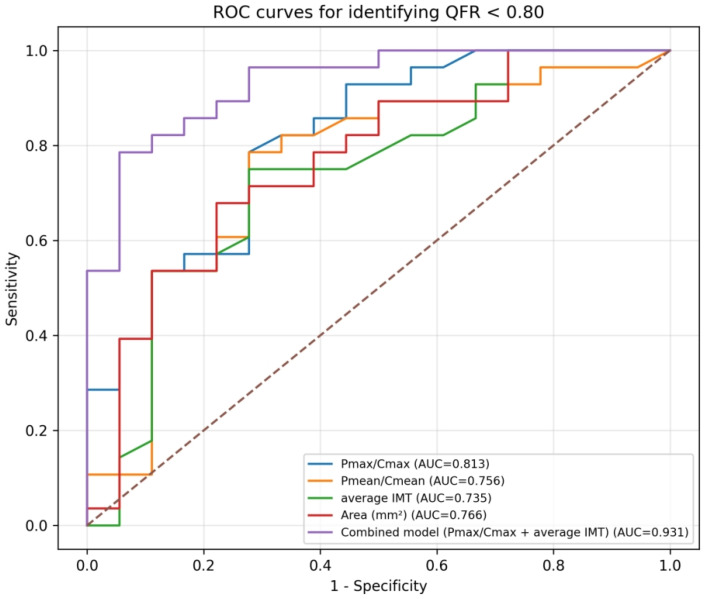
ROC curves of Pmax/Cmax, Pmean/Cmean, average IMT, Area, and the combined model for identifying QFR < 0.80. Circles indicate the optimal cutoff points determined by the maximum Youden index.

A combined probability derived from the multivariable logistic regression model including Pmax/Cmax and average IMT showed improved discriminative performance, with an AUC of 0.931 (95% CI: 0.845–0.989). The AUC, optimal cutoff value, Youden index, sensitivity, and specificity for each ROC analysis are summarized in Table 5 and the ROC curves for Pmax/Cmax, Pmean/Cmean, average IMT, plaque area, and the combined model are shown in Figure 2.

## 4. Discussion

In this preliminary prospective study of patients with suspected stable CAD, carotid CEUS-derived quantitative and morphologic plaque characteristics were associated with coronary functional significance assessed by QFR. We found that: patients with QFR < 0.80 exhibited greater carotid plaque burden and enhanced CEUS-related plaque enhancement signals, as reflected by higher average IMT, larger plaque area, and increased plaque-to-lumen enhancement ratios (Pmax/Cmax and Pmean/Cmean). Pmax/Cmax and average IMT were independently associated with functionally significant CAS in multivariable analysis, and the combination of these two markers achieved excellent discrimination for QFR < 0.80 (AUC: 0.931), outperforming each single parameter.

The observed association between carotid plaque CEUS features and the functional significance of coronary stenosis supports the concept that carotid plaque vulnerability may mirror systemic atherosclerotic activity rather than represent a purely local vascular phenomenon [10,15,16]. Carotid and coronary atherosclerosis share common pathophysiological mechanisms, including lipid accumulation, inflammatory cell infiltration, endothelial dysfunction, and progressive plaque destabilization, and intraplaque neovascularization as a mark of vulnerable plaque should be focused on [17,18]. CEUS is suited to capture this process. A systematic review showed that CEUS enhancement correlated with the presence and degree of histological intraplaque neovascularization, supporting its value as an alternative imaging marker of plaque instability [19]. Moreover, CEUS-detected carotid plaque neovascularization has been associated with significant and complex coronary artery disease as well as future cardiovascular events, suggesting that carotid CEUS may reflect the overall burden of vulnerable atherosclerosis, including within the coronary circulation [20,21]. Therefore, a higher Pmax/Cmax ratio in this study may indicate more active intraplaque microvascularization and vulnerability.

It should be emphasized that carotid CEUS and QFR evaluate different aspects of atherosclerotic disease. Carotid CEUS mainly characterizes carotid plaque morphology and intraplaque neovascularization, whereas QFR is an angiography-based physiological index reflecting the hemodynamic significance of coronary stenosis. Therefore, QFR does not provide direct information on coronary plaque composition or vulnerability. The present study should be interpreted as an investigation of the association between carotid plaque vulnerability-related features and QFR-defined functional coronary significance, rather than as a direct comparison of plaque morphology between carotid and coronary arteries.

In addition to CEUS-derived plaque enhancement, average IMT likely captures a different, but complementary, dimension of vascular disease. Carotid IMT is widely regarded as a marker of arterial wall remodeling and cumulative exposure to atherosclerotic risk factors, and increased IMT has been associated with future coronary and cerebrovascular events [22,23,24]. Previous work suggested that plaque and IMT reflect different aspects of atherogenesis and vascular risk [22,25]. This may explain why average IMT remained independently associated with functionally significant coronary disease in the multivariable model. Accordingly, the superior performance of the combined model is consistent with previous evidence showing that integrating carotid wall-thickness measurements with plaque assessment improves discrimination more than either approach alone.

QFR has emerged as an attractive, less invasive alternative to FFR for assessing coronary physiological significance [8]. However, QFR still requires coronary angiography and specialized analysis. If validated in larger studies, carotid CEUS parameters—particularly quantitative plaque enhancement ratios combined with IMT—may serve as noninvasive markers to help identify patients with functionally significant coronary stenosis. Importantly, carotid CEUS is not intended to replace QFR, FFR, coronary angiography, coronary CT angiography, or cardiac MRI. Rather, it may provide complementary noninvasive information on carotid plaque neovascularization and systemic plaque vulnerability.

Previous studies have reported associations between carotid plaque CEUS enhancement and severe coronary lesions, coronary disease burden, and future cardiovascular events. Our results extend this line of evidence by focusing specifically on coronary functional significance defined by QFR, rather than anatomic stenosis severity alone. This distinction is clinically relevant because lesion severity by angiography does not always correspond to physiological ischemia. The present study therefore adds evidence that carotid CEUS features may be linked not only to coronary plaque burden but also to functionally significant ischemic lesions.

This study has several strengths. First, the prospective design reduces some retrospective selection biases. Second, carotid CEUS and QFR assessments were performed within the same evaluation period, improving temporal comparability. Third, the use of both quantitative CEUS parameters and morphologic plaque features provides a multidimensional characterization of carotid atherosclerosis.

Several limitations should be acknowledged. First, this is a preliminary single-center study with a small sample size (n = 46), which limits statistical power and external generalizability. The findings should be interpreted as hypothesis generating and require confirmation in larger multicenter cohorts. In addition, atherosclerotic burden may not be uniformly expressed across vascular territories. Although carotid and coronary atherosclerosis share common pathophysiological mechanisms, functionally significant coronary stenosis may occur in patients without evident carotid plaque or marked intima-media thickening [18]; therefore, a negative carotid CEUS finding should not be interpreted as excluding functionally significant CAD. Second, some categorical variables had sparse cell counts, preventing stable OR estimation in logistic regression and necessitating reliance on overall contingency test *p* values for screening. Third, only the carotid plaque with the greatest plaque height was analyzed per patient, which may not fully capture total carotid plaque burden or heterogeneity. Fourth, external validation and calibration assessment of the multivariable model were not performed, and the high AUC observed for the combined model may be optimistic given the sample size. Moreover, carotid CEUS mainly evaluates carotid plaque enhancement and intraplaque neovascularization and does not directly measure coronary luminal stenosis or lesion-specific coronary flow limitation.

Multicenter studies are needed to validate these findings, define robust cutoffs for clinically usable CEUS parameters, and compare the value of carotid CEUS over conventional risk factors and standard carotid ultrasound measurements.

## 5. Conclusions

In this preliminary study, carotid CEUS-derived plaque enhancement characteristics, particularly Pmax/Cmax, together with average IMT, were independently associated with QFR < 0.80 and demonstrated good discriminatory performance for identifying functionally significant coronary stenosis. A combined model integrating these parameters achieved promising diagnostic performance, suggesting that carotid CEUS may serve as a promising noninvasive imaging window for the detection of functionally significant CAS, but it should not be considered a substitute for coronary angiography-based physiological assessment.

## Figures and Tables

**Table 1:** Baseline characteristics of the study population.

Characteristic	Overall (n = 46)	QFR ≥ 0.80 (n = 18)	QFR < 0.80 (n = 28)	*p* Value
Age, years	58.93 ± 9.27	57.17 ± 8.13	60.07 ± 9.90	0.285
Systolic blood pressure, mmHg	128.50 ± 13.64	124.67 ± 11.87	130.96 ± 14.33	0.113
Diastolic blood pressure, mmHg	77.43 ± 8.33	74.67 ± 8.34	79.21 ± 7.97	0.075
Heart rate, beats/min	66.54 ± 9.84	66.17 ± 10.76	66.79 ± 9.40	0.843
Body surface area, m^2^	1.83 ± 0.19	1.83 ± 0.26	1.84 ± 0.14	0.909
Body mass index, kg/m^2^	25.35 (23.22, 27.62)	25.76 (24.46, 27.53)	25.27 (22.74, 27.49)	0.813
HbA1c, %	6.33 ± 0.99	6.29 ± 0.87	6.35 ± 1.07	0.848
Glucose, mmol/L	6.51 (5.67, 8.91)	5.79 (5.55, 8.46)	6.89 (5.97, 9.48)	0.251
Triglycerides, mmol/L	1.31 (0.92, 2.22)	1.43 (0.92, 2.04)	1.29 (0.96, 2.40)	0.710
HDL-C, mmol/L	1.24 ± 0.34	1.33 ± 0.39	1.18 ± 0.30	0.177
LDL-C, mmol/L	2.04 (1.60, 2.73)	2.00 (1.60, 2.82)	2.23 (1.65, 2.72)	0.804
hs-cTnI, ng/mL	0.03 (0.01, 0.07)	0.02 (0.00, 0.06)	0.04 (0.01, 0.08)	0.361
NT-proBNP, pg/mL	103.50 (53.23, 185.75)	64.10 (40.12, 120.75)	118.50 (64.90, 210.00)	0.077
Male sex, n (%)	38 (82.6%)	12 (66.7%)	26 (92.9%)	**0.042**
Hypertension, n (%)	26 (56.5%)	10 (55.6%)	16 (57.1%)	1.000
Hypertension duration > 5 years, n (%)	19 (41.3%)	9 (50.0%)	10 (35.7%)	0.513
Hypertension duration > 10 years, n (%)	16 (34.8%)	7 (38.9%)	9 (32.1%)	0.879
Hyperlipidemia, n (%)	38 (82.6%)	13 (72.2%)	25 (89.3%)	0.232
Diabetes mellitus, n (%)	18 (39.1%)	4 (22.2%)	14 (50.0%)	0.115
Smoking history, n (%)	25 (54.3%)	8 (44.4%)	17 (60.7%)	0.437
Drinking history, n (%)	14 (30.4%)	3 (16.7%)	11 (39.3%)	0.194

**Table 2:** Baseline and plaque-related characteristics according to QFR category.

Variable	QFR ≥ 0.80 (n = 18)	QFR < 0.80 (n = 28)	*p* Value
**Quantitative plaque and luminal parameters**			
Mean IMT, mm	0.92 ± 0.15	1.04 ± 0.12	**0.011**
Maximum plaque height, mm	2.62 ± 0.74	2.86 ± 0.64	0.271
Plaque length, mm	11.28 ± 6.05	12.38 ± 6.36	0.558
Plaque area, mm^2^	13.68 (9.64, 18.24)	21.41 (16.31, 29.05)	**0.003**
Maximum plaque-to-carotid lumen intensity ratio (Pmax/Cmax)	0.44 (0.21, 0.67)	0.75 (0.64, 0.84)	**<0.001**
Mean plaque-to-carotid lumen intensity ratio (Pmean/Cmean)	0.37 ± 0.26	0.61 ± 0.22	**0.003**
Plaque mean intensity, dB	6.96 (3.87, 16.29)	13.50 (9.00, 19.98)	0.100
Plaque maximum intensity, dB	16.54 (12.06, 26.71)	22.61 (18.62, 25.50)	0.087
Plaque wash-in slope	1.89 (1.11, 4.23)	2.90 (1.79, 5.04)	0.317
Plaque time to peak	10.08 (8.22, 15.10)	8.63 (6.67, 13.31)	0.518
Plaque peak intensity	12.39 (8.39, 15.78)	17.03 (11.61, 19.29)	0.141
Plaque AUC	302.60 (216.51, 474.54)	344.81 (198.57, 527.71)	0.866
Plaque time from peak to half	18.02 (7.07, 20.97)	12.13 (8.34, 20.93)	0.986
Plaque rise time	6.06 (3.92, 8.98)	5.75 (2.72, 9.95)	0.845
Carotid luminal mean intensity, dB	25.75 (17.92, 51.12)	21.10 (16.81, 27.82)	0.308
Carotid luminal maximum intensity, dB	40.73 (31.15, 82.35)	29.36 (26.16, 43.75)	0.067
Carotid luminal wash-in slope	6.92 (4.87, 12.42)	4.57 (2.22, 6.01)	0.027
Carotid luminal time to peak	8.01 (6.37, 11.62)	8.19 (6.36, 11.96)	0.979
Carotid luminal peak intensity	32.52 (28.69, 77.31)	27.71 (22.49, 38.22)	0.044
Carotid luminal AUC	1128.37 (877.84, 1644.90)	805.04 (427.02, 1314.11)	0.067
Carotid luminal time from peak to half	18.08 (12.98, 24.45)	15.09 (12.95, 22.38)	0.570
Carotid luminal rise time	5.38 (4.33, 6.09)	5.66 (4.46, 7.87)	0.804
Plaque morphological characteristics			
Echogenicity of the largest plaque *	n = 17	n = 28	0.033
1	3 (17.6%)	1 (3.6%)	
2	2 (11.8%)	1 (3.6%)	
3	6 (35.3%)	4 (14.3%)	
4	6 (35.3%)	22 (78.6%)	
Missing	1	0	
Plaque margin **	n = 17	n = 28	0.004
Regular	15 (88.2%)	12 (42.9%)	
Irregular	2 (11.8%)	16 (57.1%)	
Missing	1	0	
Fibrous cap ***	n = 17	n = 28	0.006
Thick	13 (76.5%)	9 (32.1%)	
Thin	4 (23.5%)	19 (67.9%)	
Missing	1	0	
CEUS grading variables			
Plaque-RADS	n = 18	n = 28	0.036
1	2 (11.1%)	0 (0.0%)	
2	7 (38.9%)	5 (17.9%)	
3	9 (50.0%)	18 (64.3%)	
4	0 (0.0%)	5 (17.9%)	
CEUS grade (3-point scale)	n = 7	n = 21	0.931
0	0 (0.0%)	1 (4.8%)	
1	1 (14.3%)	2 (9.5%)	
2	1 (14.3%)	3 (14.3%)	
3	5 (71.4%)	15 (71.4%)	
Missing	11	7	
CEUS grade (Chinese 4-point scale)	n = 9	n = 24	0.005
0	1 (11.1%)	0 (0.0%)	
1	1 (11.1%)	3 (12.5%)	
2	7 (77.8%)	6 (25.0%)	
3	0 (0.0%)	15 (62.5%)	
Missing	9	4	

Abbreviations: QFR, quantitative flow ratio; IMT, intima-media thickness; AUC, area under the curve; CEUS, contrast-enhanced ultrasound; RADS, Reporting and Data System; P, plaque; C, carotid lumen. * Echogenicity of the largest plaque: 1 = hyperechoic, 2 = predominantly hyperechoic, 3 = predominantly hypoechoic, 4 = hypoechoic. ** Plaque margin: regular or irregular. *** Fibrous cap: thick or thin.

**Table 3:** Univariate screening for factors associated with QFR < 0.80.

Variable	OR	95% CI	*p* Value
Pmax/Cmax	4.143	1.743–9.849	0.001
Pmean/Cmean	2.887	1.395–5.977	0.004
Average IMT	2.519	1.215–5.221	0.013
Plaque area (mm^2^)	2.950	1.050–8.290	0.040
Margin (regular vs. irregular)	—	—	0.004 *
Fibrous cap (thick vs. thin)	—	—	0.006 *
CEUS grade (Chinese 4-grade system)	—	—	0.005 *
Maximum plaque echogenicity pattern	—	—	0.033 *
Plaque-RADS	—	—	0.036 *

For continuous variables, ORs were calculated per 1 standard deviation (SD) increase. Variables marked with * were evaluated using contingency-table tests (chi-square or Fisher’s exact test) due to sparse cells; therefore, ORs and 95% CIs are not reported for these variables.

**Table 4:** Multivariable logistic regression for factors independently associated with QFR < 0.80.

Variable	OR	95% CI	*p* Value
Pmax/Cmax (per 1 SD increase)	14.394	2.718–76.220	0.002
Average IMT (per 1 SD increase)	7.740	2.040–29.363	0.003

The dependent variable was QFR < 0.80 (event = 1). The multivariable model was constructed based on univariate screening, clinical relevance, and collinearity considerations.

**Table 5:** ROC analysis for identifying QFR < 0.80.

Variable	AUC	Cutoff	Youden Index	Sensitivity	Specificity
Pmax/Cmax	0.813	0.617	0.508	78.6%	72.2%
Pmean/Cmean	0.756	0.552	0.508	78.6%	72.2%
Average IMT	0.735	0.96	0.472	75.0%	72.2%
Area (mm^2^)	0.766	18.79	0.456	67.9%	77.8%
Combined model (Pmax/Cmax + average IMT)	0.931	0.706	0.730	78.6%	94.4%

## Data Availability

The datasets generated and/or analyzed during the current study are not publicly available due to patient privacy and institutional restrictions but are available from the corresponding author upon reasonable request and subject to approval by the relevant institutional ethics committee, where applicable.
